# Serum paraoxonase 1 (PON1) measurement: an update

**DOI:** 10.1186/1746-6148-10-74

**Published:** 2014-03-25

**Authors:** Jose J Ceron, Fernando Tecles, Asta Tvarijonaviciute

**Affiliations:** 1 Interdisciplinary Laboratory of Clinical Analysis INTERLAB-UMU, University of Murcia, Campus of Excellence Mare Nostrum, Murcia, Spain; 2 Departament de Medicina i Cirurgia Animals, Universitat Autònoma de Barcelona, Barcelona, Spain

**Keywords:** Dihydrocoumarin, Paraoxon, Phenyl acetate, 4-nitrophenyl acetate, Serum, TBBL

## Abstract

Paraoxonase 1 (PON1) is a widely studied enzyme based on its protective role against poisoning by organophosphate (OP) metabolites of specific OP insecticides and in vascular disease, as well as its use as biomarker of diseases involving oxidative stress, inflammation and liver diseases.

This review provides an update about the current knowledge in the field of the analytical procedures that are used for PON1 measurements. It will be specially focused on: (a) characteristics of the different substrates used for measuring PON1, with emphasis in four aspects: toxicity, polymorphism influence, rate of hydrolysis and diagnostic performance. And (b) the technical aspects of PON1 assays, in which the reagents and reaction conditions, sources of variation, quality control systems, equipment and interferences with other esterases will be discussed.

The information provided in this review can contribute to a more accurate and safe measurements of PON1 in laboratories and encourage researchers to explore the wide areas of PON1 in veterinary medicine that are still unknown.

## Introduction

Paraoxonases are a family of three enzymes called PON1, PON2 and PON3. They have multifunctional roles in various biochemical pathways such as protection against oxidative damage and lipid peroxidation, contribution to innate immunity, detoxification of reactive molecules, bioactivation of drugs, modulation of endoplasmic reticulum stress and regulation of cell proliferation/apoptosis. Since they are able to perform multiple autonomous and often unrelated functions, they are considered as “moonlighting proteins” [1]. PON1 is the most studied enzyme of the family. It is synthesized primarily in the liver and appears mainly in serum, where is associated to high-density lipoproteins (HDL) [2]. PON2 is located intracellularly and PON3, although appears also in serum, is around 2 orders of magnitude less abundant than PON1 [3].

PON1 has been widely studied in human medicine with excellent reviews produced by different research groups [4-7]. Initially the interest on this enzyme arose from the toxicological point of view, by its protective role from poisoning by organophosphate derivates. But more recently research has been focused on other clinical aspects such as protective role in vascular disease as well as its use as biomarker of diseases involving mainly three situations: (a) oxidative stress, since PON1 protects against oxidation [8]; (b) inflammation, being considered PON1 as a negative acute phase protein [9] and (c) liver diseases, because PON1 is synthesized in this organ [10]. In veterinary medicine, studies of PON1 have been traditionally focused in bovine [11,12], however in recent years it has gained interest in other species such as dogs [13,14], cats [15] and horses [16].

PON1 can be measured based on its activity by spectrophotometric assays and also can be directly quantified by immunological methods using specific antibodies [17]. The spectrophotometric assays based on the ability of PON1 to hydrolyse substrates are currently more widely used, probably due to their low cost and availability. PON1 was named paraoxonase for its ability to hydrolyze paraoxon (diethyl p-nitrophenyl phosphate, E600), the toxic oxon metabolite of parathion [18]. However PON1 is considered as a promiscuous enzyme having also the ability to hydrolyze many other substrates such as other organophosphorous compounds, non phosphorous arylesters and also lactones, which have been considered as its primary substrates [19].

PON1 can be evaluated by its *different activities*, for example its paraxonase activity (when paraoxon is used as substrate), arylesterase activity (when a non phosphorous arylester such as phenyl acetate or 4 (p)-nitrophenyl acetate is used as substrate) or by its lactonase activity (when 5-thiobutil butyrolactone –TBBL- or other lactones such as dihydrocoumarin are used as substrate). However, this terminology seems not to be totally accurate from the chemistry point of view, because “aryl” refers to any functional group or substituent derived from an aromatic ring; and therefore paraoxon could be also considered as an arylester and included in the group of the arylesterases. Maybe it would be more appropriate to use the general term “PON1 activity” that can be measured by *different substrates* such as paroxon, phenyl acetate, 4-nitrophenyl acetate, TBBL or dihydrocoumarin. These substrates (Table 1) are currently the most frequently used. However there are other substrates such as chlorpyrifos, diazinon, sarin or soman in the group of organophosphorous compounds [20] and other different lactones [19,21] that can be also employed in PON1 assays.

**Table 1:** Chemical structure of the most frequently used substrates for PON1 assays

**Paraoxon**	**Phenyl acetate**	**4-nitrophenyl acetate**	**TBBL**	**Dihydrocoumarin**
				

Currently in PON1 measurements described in literature, there is a great heterogeneity in the selection of the substrates as well in the conditions of the assays used with each substrate. This situation can lead to difficulties in the comparison of values obtained by the different laboratories, generation of inappropriate results for not using adequate analytical procedures, or problems in interpretation due to the different substrate activity polymorphisms. This report will deal with two main topics: (a) characteristics of the different substrates that can be used for measuring PON1 activity with a special focus on the most widely used and (b) the technical aspects of PON1 assays.

## Characteristics of the substrates

At this point, four characteristics (toxicity, polymorphism influence, rate of hydrolysis and diagnostic performance) of the main substrates used for PON1 measurements will be reviewed.

### Toxicity

European hazard codes for the main substrates used for PON1 measurements appear in Table 2.

– *Paraoxon*. It is very unstable and extremely toxic [6] and can cause a rapid and severe organophosphate poisoning [22]. In addition to the European hazard codes described in Table 2, paraoxon possesses the European risk statements number 26 (very toxic by inhalation), 27 (very toxic in contact with skin) and 28–50 (in which there is included that may cause cancer and hereditable genetic damage).

Paraoxon stock solutions should be handled in an air-extraction fumehood and the operator should take appropriate safety precautions such as wearing a face mask and double nitrile gloves to protect against accidental contact or inhalation of the toxic fumes. In addition it can produce pollution to the laboratory equipment, therefore dedicated sample and reagent needles should be used, and waste water receptacle as well waste disposal and pipettes should be treated with NaOH [6,22,23]. In particular paraoxon presents problems for automation in biochemical analysers, since it can contaminate them producing difficulties to perform assays for other analytes on the same instrument, and the removal of its traces requires the use of high concentration of NaOH which could damage instruments [24].

Other organophosphorous such as diazinon, chlorpyrifos, soman or sarin are also highly toxic and therefore special cautions should be taken with them.

– *Phenyl acetate*. It is considered not as toxic as paraoxon [6,25] but needs to be handled under appropriate safe conditions. It has the Xn (harmful) European hazard code and the European risk statement number 22 (harmful if swallowed).

– *4- nitrophenyl acetate*. It has been described as non toxic [26] and does not have any hazard advice according with the European hazard code.

– *5-thiobutil butyrolactone (TBBL*) is considered as not toxic [6].

– *Dihydrocoumarin*. In addition to have the Xn (harmful) European hazard code, it has the European risk statements 22–36 (which include very toxic by inhalation and very toxic in contact with skin), 37 (irritating to the respiratory system) and 38 (irritating to the skin).

**Table 2:** Toxicity of the different substrates used for PON1 measurements

	**Paraoxon**	**Phenyl acetate**	**4-nitrophenyl acetate**	**TBBL**	**Dihydrocoumarin**
Toxicity (European hazard code)	T + (very toxic) N (dangerous for the environment)	Xn (harmful)	Non toxic	Non toxic	Xn (harmful)

### Polymorphism influence

PON1 using paraoxon as substrate in human population has a polymorphism of high versus low activity. There are various genetic polymorphisms described in PON1 gene, but the polymorphism in position 192 seems to be more involved in the differences in PON1 activity between individuals [27,28]. This polymorphism has two isoforms: PON1Q and PON1R. Leading to three phenotypic groups: QQ representing low, QR intermediate and RR high enzyme activity of PON1 [18], that can produce significant differences in the values of PON1 activity between QQ and RR individuals [23].

This is a particular situation that occurs with paraoxon; since other organophosphorus such as diazinon or sarin showed a different polymorphism effect with a higher hydrolytic rate with the Q than the R allele [20]. And other substrates do not show such evident polymorphisms, for example:

– Phenyl acetate is hydrolyzed by PON1 phenotypes with comparable potency [29] (Table 3). Also ELISA and Western blot based methods utilizing PON1 antibodies confirm a high correlation between measured PON1 concentrations and activity measured with phenyl acetate [30,31]. However in a study individuals with RR had 1.46 fold higher PON1 values than QQ phenotype using phenyl acetate as substrate [23].

– 4-nitrophenyl acetate had similar values of activity for PON1 phenotypes when purified enzyme was used. Although in serum these values showed higher differences (Table 3) [29].

– TBBL has polymorphism influence, although it is lower than when paraoxon is used as substrate. In a previous report it was found a maximum difference between medians of QQ and RR isoforms of 40.65% for TBBL versus 167.82% for paraoxon [32]. In general lactones are hydrolyzed preferentially by either PON1R or PON1Q depending on their structure [33].

**Table 3:** Activities of different substrates in human serum and purified PON1 (in micromol substrate hydrolysed per min per mg of protein)

**Substrate**	**Serum**		**Purified enzyme**	
	**QQ**	**RR**	**QQ**	**RR**
Paraoxon	0.001	0.005	0.467	2.1
Phenyl acetate	1.26	1.52	845	720
4-nitrophenyl acetate	0.022	0.04	6.4	7.3
1-naphtyl acetate	0.023	0.032	<0.01	<0.01

Adapted from [29].

PON1 enzyme levels can range widely even between individuals with the same PON1 phenotype [34] and PON1 phenotypes do not influence changes in PON1 associated with some clinical conditions such as hepatic disease [27,35]. However in substrates highly influenced by polymorphisms, such as paraoxon in humans, there is a potential for obtaining false high values of PON1 in populations with high frequency of PON1R when compared with populations with high frequency of PON1Q and vice versa [4]. Ideally in these cases comparison between values of each different phenotype would be recommended instead a global comparison of values in all population, fact that would complicate the interpretation of the results [36,37].

There are not many studies about polymorphism in veterinary medicine. This is in contrast with the situation in humans, in which it is a very widely studied topic with methods (i.e. two-substrate assay) developed to explore and differentiate PON1 activity polymorphisms [18]. Rabbits possess a serum polymorphism which is located within exon 4 producing two different phenotypes (rPON1A and rPON1B) that have the same activity for phenyl acetate, but different values for paraoxon, dihydrocoumarin and other lactones [38]. And contrarily to humans, individual variations of PON1 in pigs with phenyl acetate are genetically determined, and phenotypes with high, intermediate and low PON1 activity against this substrate were described [39,40]. Also there have been evidences that variations in plasma PON1 activity in cattle could be genetically determined [41]. To the author’s knowledge no studies to fully characterize polymorphisms have been undertaken in other species. Therefore studies made with populations should be taken with caution in veterinary medicine.

### Rate of hydrolysis

In humans, rate of PON1 hydrolysis of paraoxon is lower compared with other substrates such as phenyl acetate or 4-nitrophenyl acetate (633 and 29 times higher respectively) [42] and even compared with other organophosphorus compounds such as diazoxon or chlorpyrifos oxon [43]. Similar results were obtained by other authors [26,29]. These last authors made a complete study in which the rate of hydrolysis of different substrates were compared in serum and with purified enzyme for the two different genotypes (Table 3). In this table it can be observed that most of the activity when using paraxon, phenyl acetate and 4-nitrophenyl acetate substrates in serum is due to PON1; since there is enrichment in the activity when the enzyme is purified. However, when other substrates such as 1-naphtyl acetate are used, hydrolysis in serum is probably due to other different esterases, since purified PON1 does not hydrolyse this substrate.

The low hydrolysis rate of paraoxon has lead to some authors to indicate that it could be inappropriate to call the enzyme “paraoxonase” or “organophosphatase” [29]. Even PON1 has been considered as a good example of the power of language to mislead, because the focus of the name on one aspect of the enzyme’s function, which is related with its behaviour with synthetic and exogenous substrates (organophosphorous compounds), has delayed an appreciation of other potential and perhaps more relevant roles, that are more related with natural and endogenous substrates such lactones [44].

In veterinary, although few data are available, it seems that PON1 can have a different spectrum of activities not only to different substrates but also to the same substrate, depending on the species. For example minipig, rabbit and mouse have higher rate of 4-nitrophenyl acetate hydrolysis than rat, monkey or dog (Table 4). Regarding the inter-species variations of the activities of different substrates, in general 4-nitrophenyl acetate is hydrolyzed at a higher rate than paraoxon [45,46]. And in pigs 4-nitrophenyl acetate has eight times higher rate of hydrolysis than phenyl acetate; whereas in sheep and dogs phenyl acetate is hydrolysed at a higher rate than 4-nitrophenyl acetate [41,46-48].

**Table 4:** **Rate of hydrolysis of 4-nitrophenyl acetate in different species (micromol/min/ml serum) [**[49]**]**

**Species**	**Rate**
Minipig	31.5
Rabbit	12.7
Mouse	6.8
Human	3.0
Dog	2.9
Monkey	2.7
Rat	2.5

Although the lactones are considered natural substrates, and therefore would better reflect the physiological activity of PON1, highly correlated activities were found in humans using TBBL and phenyl acetate; and both were inhibited in the same manner by selective competitive inhibitors of PON1’s activity such as 2-hydroxyquinoline and EDTA [19]. This could explain the high correlation when using phenyl acetate and TBBL observed in human studies [50], bovine [51] and dog [48]. In this later study in dogs, TBBL, phenyl acetate and 4-nitrophenyl acetate showed significant correlations with r higher than 0.85 in all cases. Maybe an explanation for these results is the fact that both the activity of PON1 against lactones and phenyl acetate or 4-phenyl acetate derivates are mediated by the same active site of the enzyme, which is different than the site where is the activity against phosphorous agents [52].

### Diagnostic performance

In studies in which different substrates have been compared, two situations can be found:

#### 

**a) Conditions or diseases in which the substrates give similar information** For example:

In human medicine decreases of similar magnitude on PON1 activity in humans with HIV using paraoxon and TBBL (1.21 versus 1.28 fold) have been found [53]. And TBBL diagnostic accuracy was similar than paraoxon to diagnose liver failure [32].

In veterinary, similar diagnostic performance was detected between dihydrocoumarin, phenyl acetate and paraoxon for diagnosis of fatty liver in cows [51].

In the conditions of the point “a” the logical approach would be to choose the substrate that would be less toxic, less expensive and readily adapted to analytical instruments and conditions of the lab.

#### 

**b) Conditions or diseases in which there can be divergences between substrates** In a human study comparing paraoxon, phenyl acetate and 4-nitrophenyl acetate, the last one was the only that showed significant changes between patients with non-end-stage chronic renal failure and healthy individuals. This substrate showed higher activities between narrower ranges of population values that could probably reflect less analytical errors [26]. In other study phenyl acetate was found to be more sensitive and specific substrate in identifying patients with chronic hepatic disease than paraoxon [27]. And a stronger association between decreased serum PON1 activity and metabolic syndrome was found in childhood obesity when TBBL was used as substrate compared to paraoxon [54].

In dogs higher significance in the differences between dogs with oxidative damage and healthy dogs was found with 4-nitrophenyl acetate compared with phenyl acetate or TBBL [48].

## Technical aspects of the assays

### Setting up the assay: reagents and reaction conditions

The principle of the spectrophotometric determination of PON1 activity is simple. The sample is mixed with a substrate dissolved in a buffer solution, and the product generated by the hydrolysis of the substrate is monitored during certain time at a linear rate and defined temperature. However there is a heterogeneicity in the concentration of reagents used and assay conditions in general.

– *Buffers.* The most used is Tris–HCl, but other buffers such as glycine have been also used [26]. The ideal pH of the reaction is in the range of 8 and 8.5 [18,55]. Since below pH 8 there is a significant decrease in PON1 activity, whereas at values higher than 8.5 the non-specific esterase activity of the albumin starts to be activated [55]. Additionally also very basic pH buffers can lead to alterations of PON1 structure and activity [56]. Therefore those assays that use values out of this pH range, as 7 or 10.5 should be interpreted with caution.

– *Substrate concentration.* There is a wide variation in the concentration of substrates used in the assays described in the literature. For example paraoxon ranges from 1 to 5 mmol/L, phenyl acetate from 0.2 to 6.64 mM, 4-nitrophenyl acetate from 0.5 to 2.5 mM (although a report indicated 27.6 mM [29]) and TBBL from 0.25 to 0.5 mmol.

The concentration of the substrate should be established and optimised together with sample volume to assure the linearity of the reaction; avoiding substrate consumption. Ideally this concentration should be high enough than Km in order to produce the maximum velocity, in which the substrate is saturating the enzyme and the activity only depends of the amount of the enzyme in the sample (Table 5). However the substrate concentration should not be too high; since further increases in substrate concentrations after these saturating conditions will not have any effect on the kinetic of the reaction, but produce higher non-enzymatic hydrolysis. In some cases saturating conditions are difficult to be reached during the limited solubility of substrates, as has been described with paraoxon [57]; therefore in this case substrate concentrations should at least assure the linearity of reaction.

**Table 5:** Km (concentration of substrate that produces half of the maximum velocity of the reaction) and wavelengths (גּ) used for different substrates of PON1

**Substrate**	**Km (mM)**	**Reference**	**גּ (nm)**
**Paraoxon**	1.2 ± 0.2	[58]	412
	1.4	[59]	405
	0.8 ± 0.1	[60]	405
**Phenyl acetate**	0.73 ± 0.08	[58]	270
	1.2 ± 0.2	[60]	270
**4- nitrophenyl acetate**	1.5 ± 0.2	[60]	405
**TBBL**	2.8	[33]	412 (detected with DTNB)
	0.27 ± 0.04	[52]	
**dihydrocumarin**	0.02	[33]	270
	0.129 ± 0.008	[60]	

Some substrates require an organic solvent to be dissolved. In these cases methanol is recommended instead acetone since it is less inhibitory for PON1. For example at final methanol concentrations of the assay between 1-2%, inhibition is negligible, whereas acetone produces a reduction of at least 25% [61]. In case of TBBL, DTNB is also added to the assay mixture in order to colorimetrically detect the released thiol moities of this substrate by PON1 activity [52].

Usually the substrates are mixed and used together with the buffer. But in some cases such as with 4-nitrophenyl acetate, to avoid spontaneous hydrolysis, it is important to remove the substrate from the working reagent buffer and prepare it in water as a separate starting reagent. This reagent remains colourless and is added to initiate the kinetic reaction [23,48].

In order to evaluate the spontaneous hydrolysis of substrates and correct the results accordingly, the use of a blank without serum is highly recommended. This value should be lower than 5% of the enzymatic hydrolysis rate [52]. If this spontaneous hydrolysis is abnormally high, new substrate should be prepared. Also the use of a blank without substrate would be recommended.

Optimal wavelengths for different substrates appear in Table 5. It is important to point out that not all standard spectrophotometers or automated analysers have available the 270 nm wavelength; so substrates with this wavelength cannot be automated or used in certain instruments. Also for wavelengths in the ultraviolet region (lower than 400 nm) quartz cuvettes will be required. Use of non-optimal wavelengths can produce increase in imprecision of the assay [23].

– *CaCl*_*2*_*.* PON1 has two calcium binding sites, one is important for the stability of the enzyme while the other one is important for the catalytic activity. Therefore, PON1 is a calcium dependent enzyme, and the addition of Ca in order to secure adequate expression of enzyme activity is recommended for the assays [62]. Usually the ranges of CaCl_2_ vary from 0.9 to 3.6 mm/L. In humans using chlorpyrifos as substrate it has been reported that PON1 activity increases when CaCl_2_ is added up to 1 mM CaCl_2_, where higher concentrations did not produce further increases in activity [55]. In some species such as rabbit low Ca concentrations in assay can have a significant influence on PON1 activity [63], so it would be recommended to optimize CaCl_2_ concentrations in the methods used.

– *NaCl*. Although used for phenotype characterization with paroxon as substrate, the addition of NaCl is not recommended for measurements at population level. Because although it produces increases in PON1 activity, it will stimulate much more the high-activity allelic form [55] and therefore would yield a higher variability in a healthy population. When using other substrates such as phenyl acetate, NaCl produces a decrease in PON1 activity [64].

– *Time of reading.* The time of readings should fulfil 3 requirements: (1) leave an initial phase without measurements in order to avoid the other esterases influence that can occur at this stage. (2) Be established in coordination with the concentration of substrate to assure that during this time a linear phase in the reaction can be produced. If substrate is used up, the enzyme’s active sites are no longer saturated and linearity can be lost; therefore substrate concentration and/or times of reaction should be reassessed. (3) Be long enough to represent adequately a phase with linearity, with various measurements during at least one minute in order to calculate the kinetics of the enzyme [25]. Usually the units in which PON1 activity is expressed are referred in minutes, being the most common unit used the U/L or mL of serum (U is the abbreviation of unit of enzyme activity, and is defined as the amount of the enzyme that catalyzes the conversion of 1 micro mole of substrate per minute).

– *Temperature.* Usually the temperatures used are in the range of 25°C to 37°C, with higher PON1 values found at 37°C. An increase of temperature by 1°C is associated with a 4.5% increase in PON1 activity using phenyl acetate as substrate [34]. Optimum temperature for paraoxon and phenyl acetate has been found in the range of 30-45°C [58]. However it would not be recommended to use temperatures higher than 37°C since it can increase substrate non-enzymatic hydrolysis.

The measurements made at room temperature may be subjected to temperature fluctuations. In order to avoid these fluctuations in case of use ELISA microplates, it is recommended to ensure temperature stabilization by setting the microplate spectrophotometer temperature to some level slightly higher than typical room temperature [34].

### Sources of variation due to the sample (dilution, anticoagulants, hemolysis, lipemia, bilirrubinemia)

In some cases a dilution of the sample is needed to maintain the reaction linearity and not produce early substrate depletion. If this dilution procedure cannot be automated the efficiency of routine analysis could be reduced [65], and also this is a source of pipetting imprecision. In addition, high dilutions can produce increases in PON1 activities in human serum (i.e. PON1 activity had an increase of 170% after 128 fold dilution) [66]. This could be particularly important in some assays using phenyl acetate in human serum, that have to dilute the samples 800 times because of high activity [67]. Maybe this effect could be reduced or neglected at lower dilutions, for example no significant increases after 1:40 dilution of dog serum were observed [48].

Ideally serum samples should be analyzed. PON1 has an absolute requirement for Ca^2+^ for activity and stability, so use of EDTA inactivates PON1 [68]. Previous studies found a significant decrease in PON1 activity when EDTA-treated plasma was used instead of serum in humans [69] and dogs [48]. Also it has been reported that lithium inhibits PON1 activity [70]. In a previous study, PON1 values with lithium heparin were around 5% lower than compared with serum [71]. This effect can be increased in cases that tubes are not filled adequately and therefore have a higher concentration of anticoagulant. Also in a different study, PON1 activity was between 21-26% higher in serum that in heparinised plasma, although enzyme activity in both types of samples were highly correlated [34]. In this study it was not specified if lithium heparin was used, and authors suggested that fibrin clots retain water, resulting in slightly higher serum concentrations. Therefore in case of use lithium-heparin, results are not equivalent to serum samples, so care should be taken when comparing data from both types of samples [71]. Caution also should be taken if sodium citrate is used as anticoagulant since it chelates calcium and also involves a dilution 1:9 of the blood [72], facts that potentially could affect PON1 activity.

The effects of the haemolysis and lipemia seem to be different depending on the assay used. For example in an study made in dogs in which different assays were compared, a significant decrease in PON1 was obtained with concentration of haemoglobin higher than 8 g/dl (that represents a very severe haemolysis) and triglycerides higher than 5 g/L when using TBBL as substrate. Similar interferences were obtained for triglycerides with an assay using phenyl acetate, however this assay was not affected by haemoglobin. And the assay using 4-nitrophenyl acetate as substrate showed decreases in PON1 when haemoglobin concentration was higher than 8 g/dl and triglycerides were higher than 1.25 g/L [48]. Overall it seems that only very severe haemolysis affects PON1 activity; similarly to described in humans where no substantial interferences by haemoglobin up to 6 g/L were found using TBBL [32]. This report also concluded that lipidemia and jaundice do not appear to have major influence on the TBBL assay.

### Quality control systems

To the author’s knowledge there is no available control serum for PON1 measurements. An alternative approach can be the use of frozen aliquots of serum as control. Ideally two serum pools, one with values inside the reference range and one with low values of PON1, from the species to be measured, could be used as controls. These control samples could be analysed just before the beginning, at the end and at intermediate points, in case of analyse a high number of samples. In case of human serum, pools of different phenotypes (QQ, QR and RR) at different activity ranges have been used for quality control [23].

Regarding the stability of these samples, in a study made in humans it was found that PON1 is stable during two years at -80°C when phenyl acetate, diazoxon and chlorpyrifos-oxon where used as substrates, but with paraoxon as substrate a decrease of 0.05% per day of storage was found [34]. In the same study, after 5 years of storage at -80°C, the enzyme activities were 17.1%, 39.4% and 37.6% lower for phenyl acetate, chlorpyrifos-oxon and paraoxon respectively and 22.9% higher for diazoxon. And no significant changes with any of the substrates after multiple freeze-thaw cycles (up to 4) in samples stored 2 years at -80°C were found.

### Equipment

PON1 assays can be measured using different equipments such as single sample spectrophotometers, microplate readers or automated analysers.

Automated analysers allow high-throughtput measurements, however they have limitations in two situations: (a) some of them do not have available the wavelengths required for the measurement of selected substrates, (b) contamination can occur when paroxon or other organophosphorous compounds are used as substrates as previously discussed. Also in case of TBBL, a solution needs to be prepared in acetonitrile for its disolution, reaching in reaction mixtures a concentration of around 1%, which can produce carryover contamination by acetonitrile and may interfere with several routine chemistries [6].

For those substrates that can contaminate automated analysers or that need wavelengths that are not included in conventional analysers, microplate-based methods could be an alternative allowing high-throughput measurement of PON1 activity. For example human assays using paraoxon, diazinon, phenyl acetate or TBBL as substrates [18] and dogs assays for phenyl acetate or TBBL [48] have been adapted to microtiter 96-well plate systems. These analyses can be performed semi-automatically, are fast and facilitate a relatively high analytical throughput [6]. In case of using paraoxon with these systems, substrate contamination should be removed by immersion of plates and pipette tips in 2 mol/L NaOH [24]. And in case of using TBBL, the samples should be analyzed in the same batch in order to avoid the high interassay variations of this assay that can reach up to 17% both in dogs [48] and humans [32].

### Interferences with other esterases

Most of the substrates used for PON1 measurements are esters (i.e. paraoxon is an organophosphorous ester and the lactones are cyclic esters) that are hydrolyzed by this enzyme. However in plasma there are other esterases such as albumin, cholinesterases (acetyl and/or butyril) or carboxylesterase that can carry out ester hydrolysis [73,49]. Also carbonic anhydrase, alpha-chymotrypsin [74] and lipase [56] can hydrolyse ester substrates. All these esterases should be taken in consideration since they could potentially interfere in the determinations of PON1.

– The effect of albumin is particularly important at pH values higher than 8.5, where the non-specific esterase activity of the albumin starts to be activated [55].

– It was described that acetylcholinesterase does not affect PON1 activity [56]. However in pigs butyrilcholinesterase (BChE) can hydrolyse PON1 substrates such as phenyl acetate; and although PON1 is 100 times more active toward this substrate than cholinesterase, this activity can be apparent in cases of pigs with very low PON1 activity [39,40]. In humans the contribution of the hydrolysis of phenyl acetate by BChE is about 5% of the total activity in serum [29].

– Human, monkey, dog or minipig plasmas do not contain any carboxylesterase, however this esterase should be taken in consideration in other species such as rabbit, mouse and rat in which it is the major component of the plasma [49].

– Carbonic anhydrase seems not to be at high concentrations in serum since is a tisular enzyme [75].

– Alpha-chymoptrypsin activity was described to be low in the phase of the reaction that usually is taken for PON1 measurements [74].

– We could not find any report about the effect of lipase in the substrates used for PON1 activity. However in situations where lipase is high such as pancreatitis, PON1 is low, therefore it seems that lipase would not have a major effect in this enzyme [76,77].

The activity of esterases different than PON1 can cause a non-linear phase in the reaction that usually occurs as an early burst in the first 15 to 30 seconds of the assay, but in some cases it can last until 15 minutes. For example using paraoxon as substrate, it was found that 10% of the samples assayed showed nonlinear kinetics during the first 15 min of the assay, linearity being established only after this time [78]. It is not clear which esterase/s produce this reaction since albumin was considered [24], but other authors pointed out that albumin preparations can be contaminated with BChE that would produce the reaction [79,80]. Also alpha-chymotrypsin produces an initial and rapid hydrolysis of the esters substrates [74].

Although there is a paucity of data about the magnitude of the hydrolysis of the different substrates by other esters different than PON1 that can appear in serum, it was described that PON1 splits esters of phenols at a higher rate than any other esterase type [41]. In this line, when using 4-phenyl acetate, it seems that there is no major effect of other non PON1-esterases in man, cow, sheep and goat, since no decrease in activity was detected after incubation of the sample with low concentration of paraoxon that would inhibit other estereases different than PON1. Also minor amounts of esterases different than PON1 appeared in rabbit and cat. However, horses have a significant amount (around 1/3 of the total activity), and rat, guinea pig and duck have a predominance of other esterases in serum [45].

Two strategies have been recommended to reduce the effect of the non-specific activity of esterases different than PON1:

– Data collection of readings should be made when the linear phase of the reaction starts, which usually occurs after some period of time. But it should not be done at zero time because it can lead to non-linear kinetics due to the activity of other esterases and overestimation of PON1 activity.

– The measurements should be performed at pH not higher than 8.5 since albumin does not have esterase activity at these pHs [55].

## Conclusions and general recommendations

Although there is still a need of more knowledge in this subject, from a general point of view, in case of PON1 measurements it could be recommended:

1. Try to select the most appropriate substrate in terms of clinical sensitivity-specificity, less toxicity and less polymorphism influence. At this point much more works about clinical sensitivity-specificity of different substrates in different species and clinical situations, as well the influence of polymorphism in these substrates (with the potential development of two-substrate assays for evaluating possible polymorphisms) would be welcome.

2. Set up adequate conditions for enzymatic reactions to avoid situations that can negatively affect measurements such as: very high pHs, too high substrate concentrations, inadequate wavelengths, or measurement in the non linear phase or the reaction.

Possibly in the future, the use of assays that can directly quantify the amount of PON1 would provide additional information to the spectrophotometric assays, as occurs in humans. For example in metabolic syndrome the finding of unmodified PON1 protein concentration and decrease in PON1 activity supports that PON1 is inactivated by oxidized lipids [54].

We hope that the information provided in this review can contribute to a more accurate and safer measurements of PON1 in laboratories already measuring or that are planning to set-up this assay in the future. It could also help to a more homogenous measurement of this enzyme and therefore the possibility of comparing values between different laboratories; since it has been observed a high reproducibility of these assays at different laboratories using the same analytical conditions and standardized protocols [34]. There is certainly a need of further studies in order to elucidate the role of PON1 in many clinical conditions related with oxidative stress and inflammation, as well its use of biomarker for diagnosis, monitoring and treatment of these diseases. And overall this review could encourage researchers to explore the wide areas of PON1 in veterinary medicine that are still unknown.

## Competing interests

The authors declare that they have no competing interests.

## Authors’ contributions

All listed authors contributed equally to the preparation and writing of the review. Also they read and approved the review prior to submission.
